# Companion dogs show signs of jealous behaviour toward non-living agents

**DOI:** 10.1038/s41598-025-86821-2

**Published:** 2025-01-24

**Authors:** Judit Abdai, Beatrix Laczi, Fabio F. Agostinho, Ádám Miklósi

**Affiliations:** 1 Department of Ethology, Eötvös Loránd University, Budapest, Hungary; 2 ELKH-ELTE Comparative Ethology Research Group, Budapest, Hungary; 3 University of Évora, Évora, Portugal

**Keywords:** Animal behaviour, Social evolution

## Abstract

Dogs engage in social interactions with robots, yet whether they perceive them as social agents remains uncertain. In jealousy-evoking contexts, specific behaviours were observed exclusively when dogs’ owners interacted with social, rather than non-social rivals. Here, we investigated whether a robot elicits jealous behaviour in dogs based on its level of animateness. First, dogs observed a robot either moving mechanistically, or showing animate motions in a non-interactive, or interactive manner towards a human. Then, in consecutive trials the owner interacted with a rival dog, the robot, and read a magazine, while ignoring the subject. Dogs displayed more owner-oriented behaviours, interaction interruption, vocalization and snapping when the owner attended the rival dog compared to reading. Dogs showed intermediate interest toward the robot but snapped more toward the robot than the magazine. Thus, dogs adjust their jealous behaviour to the actual rival, and indirect, brief experience is sufficient to consider the robot as distinct from typical inanimate objects.

**Supplementary Information:**

The online version contains supplementary material available at 10.1038/s41598-025-86821-2.

## Introduction

Jealous behaviour arises when a significant relationship with a partner is endangered by the involvement of a third party (rival)^1–3^. Jealous individuals typically exhibit behaviours that (1) draw the partner’s attention toward themselves, (2) disrupt the interaction between the rival and the partner, and (3) aim to remove the rival^4–6^. It is anticipated that these behaviours only emerge in the presence of a potential social rival and are not displayed when the partner engages with an inanimate object.

Primarily explored in humans, this phenomenon appears to manifest even in infants below the age of one year^7,8^. Since social relationships are susceptible to jeopardy in non-human animals, jealous behaviour may also emerge in other social species. Given the functional similarity between dog-owner and mother-offspring attachment^9,10^, it is conceivable that dogs (*Canis familiaris*) might also exhibit jealous behaviour if they perceive their bond with their owner to be at risk.

A handful of studies have collected evidence suggesting that dogs may exhibit jealous behaviour, although the results are mixed^6,11–13^. In these studies, the owner or a stranger attend to another dog (fake or real), or in control groups they pet an unfamiliar object or read from a newspaper. Some researchers found that dogs were more attentive to both the owner and the rival in case of ‘social’ rivals compared to the control objects in a jealousy-evoking situation^12–14^, the peak force of pulling the leash was higher when the owner attended a fake dog^11^, and also dogs displayed more blocking behaviours when the owner interacted with a fake dog but not when a stranger did^15^. However, Prato-Previde et al.^14^ found that dogs were not only attentive when a fake dog was handled by the owner, but also when the stranger did so, which is not consistent with jealous behaviour. Although Harris and Prouvost^12^ found more snapping in case of the fake dog than the other objects, Prato-Previde et al.^14^ reported no bite attempts and only sporadic chewing of the fake dog and objects. Further, Harris and Prouvost^12^ reported more whining in case of the fake dog than in case of the magazine, but Prato-Previde and colleagues^13,14^ found that only a few dogs vocalized, and also that frequency of stress-related behaviours was low.

Abdai et al.^6^ (whose method we follow in the present study) reported that dog subjects interrupted more frequently the interaction between their owner and social rivals (both familiar and unfamiliar dogs) in comparison to interactions with non-social ones (familiar and unfamiliar object; non-moving remote-controlled car and magazine). Additionally, dogs displayed more owner-oriented behaviours in the presence of a familiar dog compared to non-social partners. Behaviours oriented at rivals were primarily observed towards the unfamiliar dog. These observations implied that dogs’ behaviour was modulated by the type of the rival, and there was a tendency for jealous behaviours to emerge when the owner was interacting with social beings. Although it may be disputed whether dogs’ behaviour was related to jealous behaviour per se, importantly, some behaviours were characteristically more frequent in the case of the dog partners compared to when inanimate objects were used as partners.

It should be noted that other than Prato-Previde et al.^13^ and Abdai et al.^6^, all researchers applied a fake dog as a substitute for a real dog to elicit jealous behaviour. Although an inanimate fake dog provides higher controllability over the (potential) rival’s behaviour, it has also many disadvantages. It is very likely that the dog subjects do not regard it as a true rival because although they have dog-like physical appearance, they lack all other dog-like characteristics (e.g., motion, olfactory cues, vocal communication, etc.). Thus, it is difficult to truly compare the results of these studies, as they might measure different phenomena.

Human studies show high individual variability in behaviour when it comes to jealousy (e.g^16^), and Prato-Previde et al.^13^ also reported that dogs’ reaction vary in this situation. They found that some dogs passively monitor the owner-rival interaction whereas others react actively, interrupting the interaction and staying close to, or acting on the owner. Such individual differences can make it difficult to clearly identify jealous behaviour. Although current findings on the presence of jealous behaviour in dogs are contradictory, some dog behaviours in this context appear to vary depending on whether the owner is attending to a social or non-social test partner. Notably, this difference is observed even when the social partner (familiar dog) is present in the room while the owner interacts with a non-social object. This suggests that dogs do not simply react to the presence of another dog, but rather to the specific nature of the owner’s interaction with the test partner^13^.

Dogs’ tendency to differentiate between social and non-social rivals within these jealousy-inducing scenarios provides an avenue to explore the influence of behaviour in identifying an unfamiliar entity as a potential social companion. Previously, in a series of studies, we employed artificial agents (that appeared) capable of autonomous motion yet lacked an organism-shaped embodiment (Unidentified Moving Object, UMO) as potential social partners for dogs. Our observations revealed that dogs engaged in a range of interactions with the UMO, even after a brief initial encounter lasting between 15 and 45 min (e.g^17–20^). Importantly, dogs only displayed preference or social(-like) behaviour toward the UMO, if the UMO displayed animate (e.g., self-propelledness) or interactive (e.g., helping) behaviour, but not when the UMO displayed inanimate or mechanistic motion (animate vs. inanimate:^21,22^; interactive vs. mechanistic motion:^18,19,23^).

Our objective was to find out whether jealous behaviour emerges towards the UMO within a comparable context as used by Abdai et al.^6^, and thus we utilised the “jealousy-evoking” framework to identify whether dogs’ behaviour toward the UMO is consistent with behaviours displayed toward familiar social or non-social agents/objects. We also examined the extent to which the UMO’s degree of interactivity impacts dogs’ jealousy-like behaviour. The experiment consisted of three phases. In the *Introduction phase*, the UMO moved around the room once and then dogs could explore it without the UMO reacting to their behaviour. In the *Observation phase*, we manipulated the complexity and interactivity of the UMO’s behaviour. Thus, dogs were exposed to the UMO exhibiting either mechanistic, animate/goal-directed but non-interactive, or interactive behaviours (between-subject design). In the *Jealousy-evoking phase*, the dogs were exposed to scenarios in which their owner exclusively interacted with a fellow dog from the household, the UMO, and a magazine (within-subject comparison) in three consecutive trials (counterbalanced order).

Our hypothesis posited that dogs recognize the UMO as an animate/social entity (akin to a potential rival) and consequently display jealous-like behaviours (e.g., interrupting the owner-rival interaction) parallel to their responses towards a familiar dog. Furthermore, we anticipated that their behaviour would diverge significantly from that demonstrated when the owner interacted with a magazine. Specifically, we postulated that these jealousy-like behaviours would predominantly manifest following exposure to the UMO exhibiting animate/social behaviour (either non-interactive or interactive) during the Observation phase, but not when the UMO displayed mechanistic motion. Alternatively, we expected that animate/goal-directed motion without demonstrating the capability of social interaction (that is, the UMO capable of engaging with a human) may not be sufficient to elicit jealous behaviour toward the UMO.

## Results

### Observation phase

Before the Jealousy-evoking phase, dogs observed the behaviour of the UMO: in the Introduction phase, the UMO moved on a winding or circular route, and in the Observation phase, the UMO’s behaviour differed according to the specific group dogs were assigned to. In the Interactive UMO group, an unfamiliar human (Experimenter 1, E1) gave commands to the UMO and when the UMO ‘obeyed’, E1 petted and praised the UMO. In the Non-interactive UMO group, both E1 and the UMO carried out the same behaviours as in the first group, but the UMO did it in a reverse order; there was no interaction between E1 and the UMO. In the Mechanistic UMO group, the UMO moved around the room in a circular route, independently from E1 who moved on a winding route. Here, we tested whether dogs observed the demonstration long enough to gather sufficient information about the specific behaviour of the UMO.

Dogs’ looking duration toward the demonstration differed in the three groups (LM, LRT: $\:{\chi\:}_{3}^{2}$ = 10.152, *p* = 0.006). Dogs looked at the demonstration significantly longer in the Interactive and Non-interactive UMO groups, compared to the Mechanistic UMO group (Interactive vs. Mechanistic UMO: *β* ± SE = 11.730 ± 3.950, *p* = 0.012; Non-interactive vs. Mechanistic UMO: *β* ± SE = 10.050 ± 3.950, *p* = 0.036), but no difference was found between the two former groups (Interactive vs. Non-interactive UMO: *β* ± SE = 1.680 ± 3.950, *p* = 0.905). Considering that in the Mechanistic UMO group the motion of both E1 and the UMO was repetitive, this may not be surprising. However, it should be noted that on average dogs looked at the demonstration for more than 50% of the time in all groups (mean (%) ± SD: Interactive UMO 80.10 ± 8.66; Non-interactive UMO 78.41 ± 9.86; Mechanistic UMO 58.36 ± 16.98).

### Jealousy-evoking phase

All dogs encountered three potential rivals (hereafter ‘test partners’) in three consecutive trials (order of the test partners was counterbalanced between dogs). The owner attended exclusively to the other dog from the same household (Dog condition), the UMO (UMO condition), or he/she read from a magazine (Reading condition). We assessed dogs’ behaviours toward the owner, test partner or their interaction itself, and attempts to interrupt the interactions (see below in details).

#### Principal component analysis

We performed principal component analysis (PCA) on the duration of looking at, and orienting toward the owner, test partner and their interaction, and time spent near the owner (see Table 1 and the Methods section). Based on the parallel analysis, we retained three components described as Interaction- (PC1), Owner- (PC2), and Test partner-related (PC3) behaviours (see Table 1).

**Table 1:** Result of the PCA

Item (durations)	Interaction-related behaviour	Test partner-related behaviour	Owner-related behaviour
Orient toward interaction	**0.895**	0.034	0.036
Being near owner	**0.681**	0.121	0.479
Looking at interaction	**0.788**	− 0.084	− 0.359
Looking at test partner	0.034	**0.931**	− 0.094
Orienting toward test partner	− 0.040	**0.932**	0.040
Looking at owner	− 0.097	0.002	**0.831**
Orienting at owner	0.034	− 0.079	**0.799**
Cronbach’s alpha	0.708	0.846	0.597
Explained variance	27.1%	25.2%	24.3%

In each component, we highlighted with bold the loading of those items that significantly contributed to the component

**Fig. 1:**
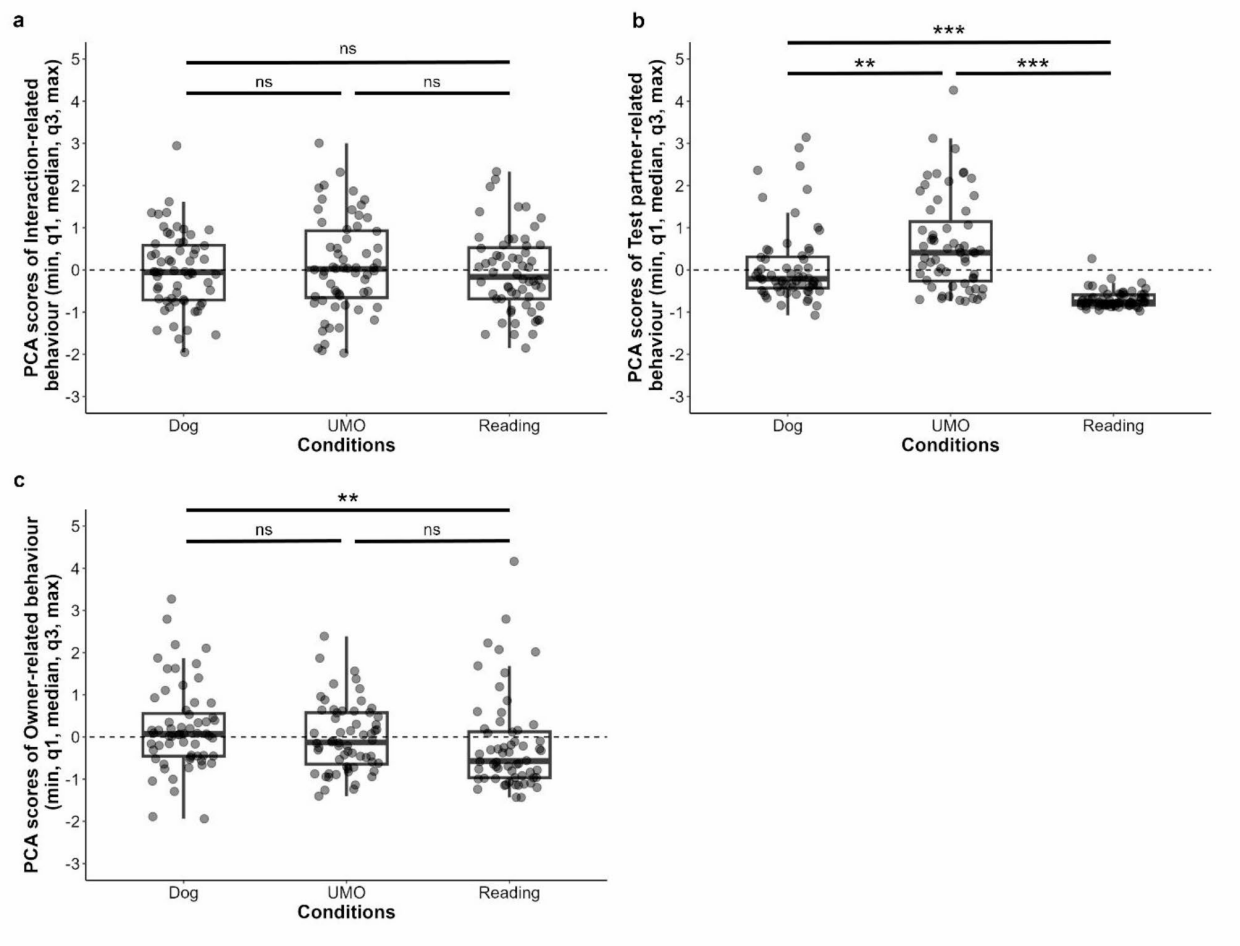
Influence of Condition on the scores of the three principal components. PCA scores of the **a** interaction-related behaviour, **b** test partner-related behaviour, and **c** owner-related behaviour, based on the condition. *ns* non-significant, ** *p* < 0.01, *** *p* < 0.001.

In the case of the Interaction-related behaviour, neither the condition by group interaction (LMM, LRT: $\:{\chi\:}_{4}^{2}$ = 6.635, *p* = 0.156), nor the group ($\:{\chi\:}_{2}^{2}$ = 0.964, *p* = 0.618) or the condition ($\:{\chi\:}_{2}^{2}$ = 1.544, *p* = 0.462) (Fig. 1a) had an effect on the component. Further, the trial number by group ($\:{\chi\:}_{4}^{2}$ = 8.558, *p* = 0.073) or the trial number by condition ($\:{\chi\:}_{4}^{2}$ = 4.263, *p* = 0.372) interactions, or trial number ($\:{\chi\:}_{2}^{2}$ = 1.443, *p* = 0.486) did not influence the component.

The condition by group interaction did not influence the Test partner-related behaviour (Box-Cox transformation—LMM, LRT: $\:{\chi\:}_{4}^{2}$ = 6.123, *p* = 0.190), neither did the group ($\:{\chi\:}_{2}^{2}$ = 4.665, *p* = 0.097), but we found the significant main effect of condition ($\:{\chi\:}_{2}^{2}$ = 110.440, *p* < 0.001). More Test partner-related behaviour occurred in the UMO than in the Dog or Reading conditions (Dog vs. UMO: *β* ± SE = 0.089 ± 0.027, *p* = 0.003; UMO vs. Reading: *β* ± SE = − 0.331 ± 0.027, *p* < 0.001), and also in the Dog condition than in the Reading (Dog vs. Reading: *β* ± SE = − 0.242 ± 0.027, *p* < 0.001) (Fig. 1b). Again, trial number by group ($\:{\chi\:}_{4}^{2}$ = 0.397, *p* = 0.983) or trial number by condition ($\:{\chi\:}_{4}^{2}$ = 1.143, *p* = 0.888) interactions, or trial number ($\:{\chi\:}_{2}^{2}$ = 1.214, *p* = 0.545) did not influence the component.

Regarding the Owner-related behaviour, we found that the condition by group interaction did not have an effect (LMM, LRT: $\:{\chi\:}_{4}^{2}$ = 2.227, *p* = 0.694), and dogs’ behaviour did not differ in the three groups ($\:{\chi\:}_{2}^{2}$ = 1.163, *p* = 0.559). However, results show that dogs’ Owner-related behaviour was different based on the condition ($\:{\chi\:}_{2}^{2}$ = 10.502, *p* = 0.005). Dogs displayed more Owner-related behaviour when the owner attended to the other dog, compared to the magazine (Dog vs. Reading: *β* ± SE = 0.393 ± 0.120, *p* = 0.004), but there was no significant difference between the Dog and UMO (Dog vs. UMO: *β* ± SE = 0.227 ± 0.120, *p* = 0.146) or the UMO and Reading conditions (UMO vs. Reading: *β* ± SE = 0.166 ± 0.120, *p* = 0.354) (Fig. 1c). Neither trial number by group ($\:{\chi\:}_{4}^{2}$ = 7.311, *p* = 0.120) or trial number by condition ($\:{\chi\:}_{4}^{2}$ = 9.040, *p* = 0.060) interactions had an effect, nor trial number ($\:{\chi\:}_{2}^{2}$ = 1.472, *p* = 0.479).

#### Frequency of interruption, snapping and vocalization

**Fig. 2:**
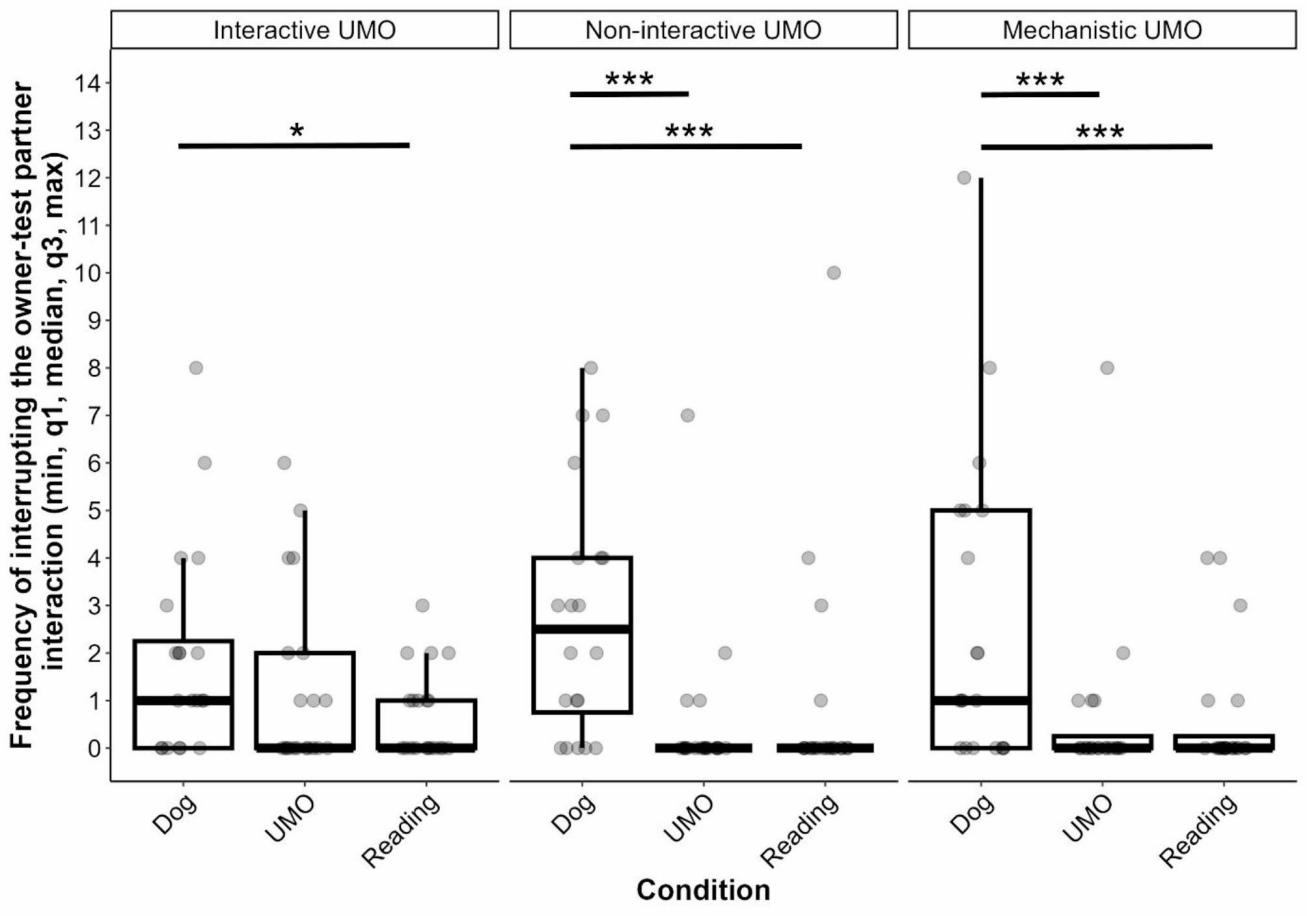
Frequency of interrupting the interaction between the owner and one of the test partners in the three groups. *** *p <* 0.001, * *p <* 0.05

We found that the two-way interaction of group and condition had a significant influence on the frequency of attempts to interrupt the interaction between the owner and the test partner (GLMM with negative binomial distribution, LRT: $\:{\chi\:}_{4}^{2}$ = 10.506, *p* = 0.033) (Fig. 2). For the detailed results of the pairwise comparison, see Table 2. In the Interactive UMO group, dogs tried to interrupt the interaction more often when the owner interacted with the other dog, compared to when they read from the magazine, but interestingly, there was no significant difference between the UMO and Dog, or the UMO and Reading conditions. In the other two groups, subjects’ attempt to interrupt the interaction was more frequent in the Dog condition than in the UMO or Reading conditions, but we found no difference between the UMO and Reading conditions. Pairwise comparison showed no difference between the groups in the three conditions.

The trial number also had a significant effect on the frequency of interrupting the interaction ($\:{\chi\:}_{2}^{2}$ = 8.924, *p* = 0.012), that is, dogs tried to interrupt the interaction more often in the first than in the third trial (Trial 1 vs. 3: *β* ± SE = 0.569 ± 0.206, *p* = 0.016), but no other difference was found (Trial 1 vs. 2: *β* ± SE = 0.446 ± 0.206, *p* = 0.078; Trial 2 vs. 3: *β* ± SE = 0.123 ± 0.227, *p* = 0.850). Importantly, this was irrespective of the specific condition (Condition x Trial: $\:{\chi\:}_{4}^{2}$ = 4.560, *p* = 0.336) or the group (Group x Trial: $\:{\chi\:}_{4}^{2}$ = 1.650, *p* = 0.800).

**Table 2:** Effect of Group X Condition on the frequency of interrupting the owner-test partner interaction; pairwise comparison (Tukey correction)

Group	Condition	β ± SE	*p* value
Between conditions			
Interactive UMO	Dog vs. UMO	0.404 ± 0.306	0.384
	UMO vs. Reading	0.499 ± 0.371	0.370
	**Dog vs. Reading**	**0.903 ± 0.357**	**0.031**
Non-interactive UMO	**Dog vs. UMO**	**1.835 ± 0.391**	**< 0.001**
	UMO vs. Reading	− 0.311 ± 0.440	0.760
	**Dog vs. Reading**	**1.525 ± 0.350**	**< 0.001**
Mechanistic UMO	**Dog vs. UMO**	**1.541 ± 0.377**	**< 0.001**
	UMO vs. Reading	0.042 ± 0.454	0.995
	**Dog vs. Reading**	**1.583 ± 0.380**	**< 0.001**
Between groups			
Interactive vs. Non-interactive UMO	Dog	− 0.392 ± 0.436	0.641
Interactive vs. Mechanistic UMO		− 0.099 ± 0.436	0.973
Non-interactive vs. Mechanistic UMO		0.293 ± 0.435	0.779
Interactive vs. Non-interactive UMO	UMO	1.039 ± 0.542	0.134
Interactive vs. Mechanistic UMO		1.038 ± 0.536	0.123
Non-interactive vs. Mechanistic UMO		− 0.002 ± 0.591	1.000
Interactive vs. Non-interactive UMO	Reading	0.230 ± 0.546	0.907
Interactive vs. Mechanistic UMO		0.581 ± 0.570	0.565
Non-interactive vs. Mechanistic UMO		0.351 ± 0.571	0.812

Significant variables are highlighted in bold

**Fig. 3:**
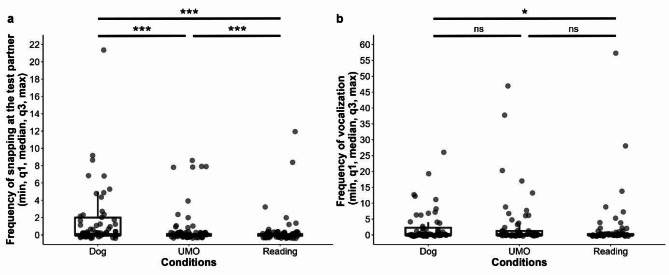
Frequency of **a** snapping at the test partner and **b** vocalizing during the three conditions. *** *p <* 0.001, * *p <* 0.05

Regarding the frequency of snapping at the test partner, dogs’ behaviour differed in the three conditions (GLMM with negative binomial distribution, LRT: $\:{\chi\:}_{2}^{2}$ = 14.483, *p <* 0.001), but not between the groups (Group x Condition: $\:{\chi\:}_{4}^{2}$ = 3.071, *p* = 0.546; Group: $\:{\chi\:}_{2}^{2}$ = 1.060, *p* = 0.589). Pairwise comparison showed that dogs snapped more at the other dog than at the UMO or magazine (Dog vs. UMO: *β* ± SE = 0.882 ± 0.009, *p* < 0.001; Dog vs. Reading: *β* ± SE = 1.796 ± 0.009, *p* < 0.001), but they also snapped more at the UMO than at the magazine (UMO vs. Reading: *β* ± SE = 0.914 ± 0.012, *p <* 0.001) (Fig. 3a). The frequency of snapping at the partner did not change over trials (Trial x Group: $\:{\chi\:}_{4}^{2}$ = 1.803, *p* = 0.772; Trial x Condition: $\:{\chi\:}_{4}^{2}$ = 4.072, *p* = 0.396; Trial: $\:{\chi\:}_{2}^{2}$ = 1.493, *p* = 0.474).

In the case of frequency of vocalization, we also found the significant main effect of Condition (GLMM with negative binomial distribution, LRT: $\:{\chi\:}_{2}^{2}$ = 7.247, *p* = 0.027); however, not the Group (Group x Condition: $\:{\chi\:}_{4}^{2}$ = 9.002, *p* = 0.061; Group: $\:{\chi\:}_{2}^{2}$ = 2.094, *p* = 0.351). Dogs vocalized more often when the owner attended the other dog compared to when they read from the magazine (Dog vs. Reading: *β* ± SE = 0.932 ± 0.360, *p* = 0.026), but we did not find significant differences between the UMO and Dog (Dog vs. UMO: *β* ± SE = 0.190 ± 0.336, *p* = 0.839), or the UMO and Reading (UMO vs. Reading: *β* ± SE = 0.741 ± 0.364, *p* = 0.104) conditions (Fig. 3b). The trial number also had an effect on dogs’ vocalization ($\:{\chi\:}_{2}^{2}$ = 8.387, *p* = 0.015), irrespectively of the Group or Condition (Trial x Group: $\:{\chi\:}_{4}^{2}$ = 6.332, *p* = 0.176; Trial x Condition: $\:{\chi\:}_{4}^{2}$ = 8.318, *p* = 0.081). Analysis showed no difference in the frequency of vocalization in Trial 1 compared to Trial 2 or 3 (Trial 1 vs. 2: *β* ± SE = -0.448 ± 0.336, *p* = 0.376; Trial 1 vs. 3: *β* ± SE = 0.582 ± 0.366, *p* = 0.250), but dogs vocalized more often in Trial 2 than in Trial 3 (Trial 2 vs. 3: *β* ± SE = 1.030 ± 0.360, *p* = 0.012).

**Table 3:** Summary of the results based on the effect of Condition (and Group where relevant)

	Dog vs. reading	Dog vs. UMO	UMO vs. reading
Owner-related behaviours (no difference between groups)	Dog > reading	ns	ns
Test partner-related behaviours (no difference between groups)	Dog > Reading	Dog < UMO	UMO > Reading
Interaction-related behaviours (no difference between groups)	ns	ns	ns
Frequency of interruption—Interactive UMO	Dog > Reading	ns	ns
Frequency of interruption—Non-interactive and Mechanistic UMO	Dog > Reading	Dog > UMO	ns
Frequency of snapping at the test partner (no difference between groups)	Dog > Reading	Dog > UMO	UMO > Reading
Frequency of vocalization (no difference between groups)	Dog > Reading	ns	ns

Ns indicates non-significant difference between conditions; less than and greater than signs indicate that the behaviour occurred more often in one of the two conditions. Except for the frequency of interruption, there was no difference among the three groups

## Discussion

To summarize our results (see also Table 3), dogs displayed more owner- and test partner-related behaviours and also attempted to interrupt the owner-test partner interaction, snapped at the test partner and vocalized more frequently when the owner attended the other dog from the household than in the Reading condition. This is in line with previous studies^6,12–14^, indicating that under the present conditions, dogs’ behaviour is different depending on whether the potential rival is a social (dog) or non-social (magazine) partner. Dogs showed more test-partner related behaviour toward the UMO than toward either of the other partners. Also, they snapped more often at the UMO than at the magazine, but less than at the rival dog. However, dogs’ owner- and interaction-related behaviours displayed in the UMO condition, or the frequency of vocalization did not differ from either the Dog or the Reading conditions. A similar result was found regarding the interruption of the interaction, but only when the UMO previously engaged in an interaction with the experimenter (Interactive UMO group). But in the case of the Non-interactive and Mechanistic UMOs, subjects tried to interrupt the owner-UMO (and owner-magazine) interaction less frequently than during the owner-dog interaction. Thus, all behaviours (other than frequency of interruption in the Non-interactive and Mechanistic UMO groups) were displayed more in the case of the UMO than in the case of the reading, or subjects showed intermediate behaviour (differing neither from the dog or the reading conditions). Considering these findings, we propose that the indirect and short (approx. 5 min long) experience with the UMO (independently from the observed interaction) was sufficient for dogs to consider the UMO being different from a regular inanimate object, but it was not enough to fully react to it as a social agent (see below in details).

The UMO’s behaviour displayed during the Observation phase only influenced subjects’ behaviour in one instance (interrupting the interaction). Based on this, we conclude that motion alone (irrespectively of its interactivity) is sufficient to elicit discrimination from an inanimate object. However, it should be noted that although the Mechanistic UMO moved in a repetitive way^18,19^, it had several motion characteristics that might elicit animacy perception. For example, the UMO visibly started and stopped moving by its own twice in the Observation phase and four times in the Jealousy-evoking phase, and changed its direction several times^21^. Thus, it is possible that in the present context, these animate motion cues triggered some, but not all behaviours related to jealous behaviour.

Considering also the general lack of difference among groups (in contrast to the differences among the conditions), it seems that difference in the visual attention toward the demonstration during the Observation phase did not influence dogs’ behaviour. It is likely that dogs looked less at the demonstration of the Mechanistic UMO’s behaviour because due to the repetitiveness, shorter time was sufficient to collect enough information about the partner. In the other two cases, however, the UMO showed more variable behaviour, dogs having to keep their attention on its actions longer. Interestingly though, interrupting the owner-UMO interaction occurred less only in the Non-interactive and Mechanistic UMO than in the case of the dog condition, whereas the frequency of interruption was similar when the dog and the Interactive UMO were the potential rivals. This indicates that although subjects looked at the demonstration of the Mechanistic UMO’s behaviour less than the other two groups, attempts to interrupt the owner-UMO interaction relied on the interactivity of the UMO and not on the variability in its motion (or reflecting dogs’ visual interest in the specific UMO).

Our results replicate previous findings by Abdai et al.^6^ regarding dogs’ behaviour displayed when encountering the familiar dog and the magazine as potential rivals. Thus, the method applied here is appropriate to test jealous behaviour and differences in the reaction of dogs toward social and non-social agents/objects. Although in the case of most behaviours displayed by the dog, the UMO fell in-between the rival dog and the magazine, test partner-related behaviours were mainly expressed in the case of the UMO here, and in Abdai et al.^6^ towards the unfamiliar dog. For the present study, we applied the two types of rivals from the previous study that might be most and least likely to elicit the jealous behaviour, two familiar test partners. The UMO, however, is an unfamiliar partner to dogs. In future studies, familiarity of the UMO should be matched with the potential rivals to disentangle the role of familiarity.

Our subjects displayed some behavioural differences compared to other studies^7,13,14^. The more frequent vocalization or snapping could be explained by the different inclusion criteria, individual variability, or the type of social rival. Unlike in our study, where we specifically selected dogs whose owners reported jealous behaviour, other studies did not use this criterion. This might lead to higher percentage of dogs showing these behaviours in our case. This might be further enhanced by individual differences^13^. Individual variability regarding the expression and types of behaviours displayed in a jealousy-evoking context^13^, and variability in the reaction to the UMO, together might hinder to find marked differences between the three conditions (mainly in the dog-UMO and UMO-reading contrast) and based on the animacy/sociality of the UMO. We also cannot exclude, that introducing the novel agent had a general influence on dogs’ behaviour (e.g., higher distress or attentiveness) which led to marked changes in the behaviour (e.g., higher occurrence of snapping). Although in the jealousy questionnaire of Karl et al.^15^ 70% of owners reported that their dogs show jealous behaviour, including snapping at the other dog as one of the behaviours, dogs did not show such behaviour toward the fake dog during their experiment. Prato-Previde et al.^14^ also did not find snapping behaviour toward the fake dog, whereas Harris and Prouvost^12^ reported that 25% of their subjects snapped at the fake dog. Only two previous studies used real dogs as social rivals^6,13^, thus it is difficult to find a solid explanation why in our study a relatively large percentage of the dogs (50%, *N* = 30) displayed at least one snapping action in one of the conditions).

The use of fake dogs as potential rivals is widespread when investigating jealous behaviour in dogs^11,12,15,24^, but it poses several issues regarding whether dogs accept this partner as a real dog (hence a rival) (see also e.g^4,25–27^). In the case of fake dogs, researchers relied on the visual similarity (from the viewpoint of a human) as the basis of acceptance as a real dog; however, in the case of the UMO, we rely on the animate and interactive behaviour of the agent, which in previous studies (e.g^20,21^), elicited social(-like) behaviour in dogs. Systematic investigation on the role of embodiment and behaviour of an artificial partner is needed.

Overall, dogs’ behaviour in the Jealousy-evoking phase depended on the condition, rather than on the trial number, indicating that dogs did not get exhausted or experienced increased stress due to the owner’s unavailability. However, frequency of separating the owner from the test partner reduced over trials that may emerge as a result of learning since this behaviour was ineffective to change the owners’ behaviour. Future studies should investigate further how vocalization (independently of the test partner) changes during trials and its possible underlying causes.

When investigating jealous behaviour in dogs, several alternative explanations for their behaviour have been proposed. For example, dogs’ behaviour is often seen as a potential response to a social agent in their territory^28^, but we controlled for this by testing the dogs in a novel location. Although we did not test for the influence of hierarchy rank in this study, previous research found that it does not affect dogs’ behaviour in this specific context^6^. Given that the behaviours were observed only with the dog partner and, to some extent, with the UMO, but not with the non-social partner, playfulness and boredom are unlikely explanations. It is possible that subject dogs reacted to the petting, wanting to be petted themselves, but this does not rule out the possibility that their behaviour could be considered jealous. Functionally, jealousy relates to resource competition, where the resource is the owner and/or exclusive interaction with the owner. The emergence of this type of social resource competition depends on many factors, which influence whether or not the behaviour is displayed (for example, if the dog is not particularly interested in being petted at that moment).

Future studies should explore whether increased and/or direct exposure to the UMO prompts dogs to display comparable behaviour towards the UMO as they do towards another dog in the jealousy-evoking situation, and whether adding dog-like embodiment would increase the emergence of jealous behaviour. Further, it would be important to disentangle how familiarity of the specific test partners (both social and non-social) influences dogs’ behaviour, or potentially their relationship with the specific test partner. We propose that the UMO may be a good substitute for a real dog to study jealous behaviour in the future.

## Methods

### Subjects

All methods were approved by the National Animal Experimentation Ethics Committee of Hungary (PE/EA/3741-4/2016 and PE/EA/00031 − 4/2023). All methods were carried out in accordance with relevant guidelines and regulations, the experiment was performed in accordance with the EU Directive 2010/63/EU and all methods are reported in accordance with the ARRIVE guidelines. Owners provided written consent indicating voluntarily allowing their dogs to participate in the study and that the test videos can be used for publication of identifying images in an online open-access publication.

We invited dogs above one year of age from multi-dog households, in which (based on the owner’s report) at least one of the dogs display jealous behaviour when the owner attends to the other dog(s). In case more than one dog of the owner participated in the experiment, the dogs were assigned to different groups and were tested on different days (eight households). We only invited dogs that had no prior experience with remote-controlled cars and never participated in a study involving a UMO before. Dogs were semi-randomly assigned to the three groups (counterbalancing age, sex, and breed across groups), and we aimed to have 20 dogs in all groups for the final analysis. We tested 69 dogs above one year of age from multi-dog households, the owners of which indicated that the dog display jealous behaviour. We excluded three dogs because the dog did not approach the UMO following the familiarization (see below) and two dogs because the owner behaved differently with the test partners (e.g., continuously touching the other dog and the magazine, but only talking to the UMO from a distance). We further excluded one dog because the owner indicated after the experiment that the dog was recently scolded harshly by the owner in a similar situation, two dogs due to loud external noise (in one case the other dog from the household, and in another case noise from a construction in the building). One dog was excluded because the video recording from the Observation phase was missing.

The 60 dogs remaining were assigned to three different groups based on the UMO’s behaviour in the Observation phase (different breeds): (1) Interactive UMO group (*N* = 20; 10 females; mean ± SD age: 4.8 ± 2.8 years); (2) Non-interactive UMO group (*N* = 20; 10 females; mean ± SD age: 4.2 ± 2.2 years); and (3) Mechanistic UMO (*N* = 20; 9 females; mean ± SD age: 5.1 ± 2.8 years) (for more details see Supplementary Data 1).

### Groups and conditions

**Fig. 4:**
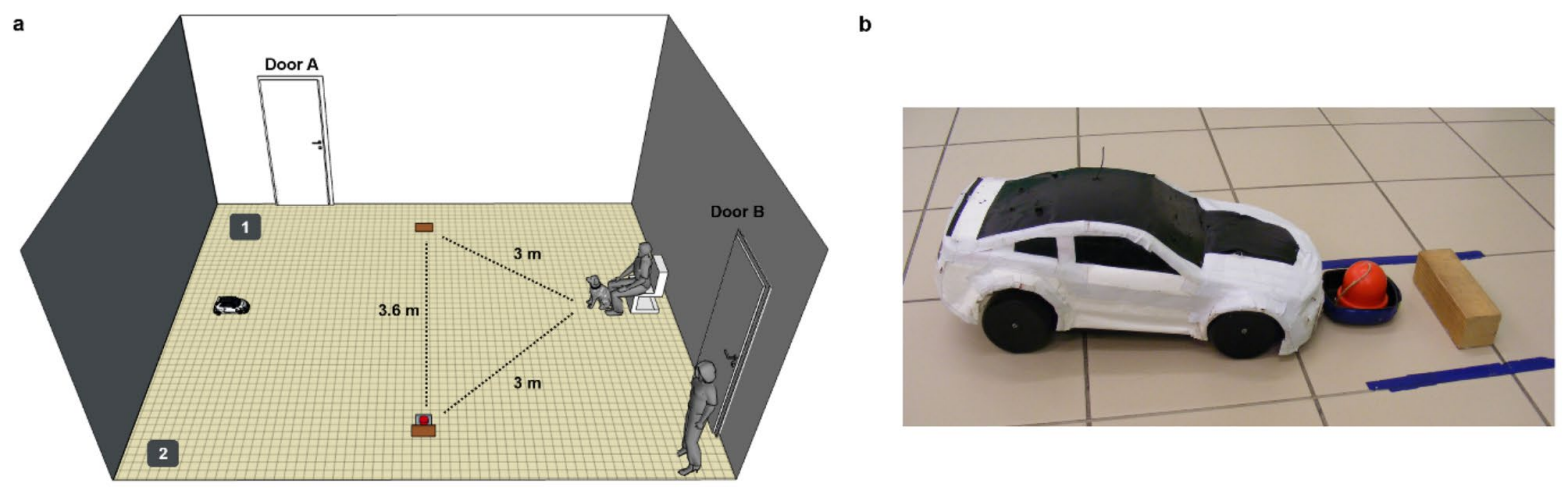
Experimental setup. **a** The arrangement of the room; 1 and 2 indicates the two places of E1 while instructing the UMO. **b**The UMO with the plate, red object, and the wooden block

We used a remote-controlled car (#32710 RTR Switch Abarth 500, basis: 28 cm x 16 cm x 13 cm) as a UMO which was covered with a white plastic cover (Fig. 4b). The UMO was controlled by Experimenter 2 (E2) from the adjacent room via live camera pictures.

In the Interactive UMO group, E1 gave different commands to the UMO (e.g., Stay!, Fetch it! ), she continuously encouraged the UMO to carry out the requested behaviour/task and when it did, E1 petted and praised the UMO. In the Non-interactive UMO group, the UMO carried out the same behaviour as the Interactive UMO, but there was no interaction between the UMO and E1. In the Mechanistic UMO group, the UMO moved around the room in a circular route, independently from E1 who moved on a winding route. Thus, (1) in the Interactive UMO group, the UMO engaged in a social interaction with a human (an interaction that can be recognized by a dog based on their daily experiences); (2) in the Non-interactive UMO group, the UMO did not display social-interactive behaviour but had complex behaviour, including behaviours that can elicit the perception of the UMO as animate^21^ and may goal-directed; whereas (3) in the Mechanistic UMO group, although the UMO’s motion contained some elements of animate motion (self-propelledness and changes in direction^21^), due to the repetition in the simple behaviour (for a relatively long time), the UMO could be viewed as a moving inanimate object (see also^29,30^ for the importance of the variability in motion, and^18,19,23^ who found that dogs display different behaviour toward a UMO depending on whether it displays interactive behaviour or merely mechanistic motion).

All dogs encountered three test partners, that is, three potential rivals: (one of) the other dog from the same household (Dog condition), the UMO (UMO condition), or the owner was reading a magazine (Reading condition). The order of conditions was counterbalanced between dogs.

### Procedure

First, dogs observed the UMO’s behaviour in the Introduction and Observation phases, carried out in a 6.27 m x 5.4 m test room at the Department of Ethology, Eötvös Loránd University, Hungary. Then after a short break (2–3 min), we carried out the Jealousy-evoking phase, during which the owner petted and talked to the test partners while ignoring the subject dog (three trials with 1–2 min breaks in-between). This phase was carried out in an adjacent, smaller test room (3 m x 5.3 m). For a video demonstration, see Supplementary Movie 1.

#### Introduction phase

Before the dog entered the room, we placed a chair next to the wall and the UMO next to the opposite side of the room. We attached two small wooden blocks (12 cm x 4 cm x 4 cm) to the ground. Next to one of the blocks there was a small plate with a red object (unfamiliar to dogs) attached to it; we counterbalanced whether the object was on the left or right side at the start (Fig. 4a).

Upon entering the room, the dog could explore the room off-leash while E1 instructed the owner. Then, the owner sat on the chair and held the dog in front of them; E1 stood in the corner on the left side of the dog (Fig. 4a). The UMO moved around the room either on a circular (mean time (s) ± SD = 33.0 ± 4.4) or a winding (mean time (s) ± SD = 51.1 ± 8.5) route before stopping again at the starting point. After the UMO stopped, the owner could release the dog and encourage the dog to approach the UMO; the UMO did not react to the behaviour of the dog. If the dog did not go to the UMO (within 10 cm), the owner and E1 went to the UMO and encouraged the dog to approach the UMO. The dog moved freely and was never forced to go near the UMO. In case the dog did not approach the UMO, it was excluded from the test. Dogs spent on average ± SD 9.3 s ± 7.0 near the UMO.

#### Observation phase

The owner sat back on the chair holding the dog in front of them, and E1 went back to the corner. The UMO started to move again on a circular/winding route, and after 10 s, E1 started to move as well. Dogs could observe one of the three demonstrations (average length (s) ± SD from the moment the UMO started to move until both E1 and the UMO left the room: Interactive UMO, 219.2 ± 23.4; Non-interactive UMO, 233.5 ± 33.4; Mechanistic UMO, 198.69 ± 25.7).

In the Interactive UMO group, E1 and the UMO engaged in an interaction, in which E1 gave different commands to the UMO which carried out the requested behaviour and then received praise from E1. E1 moved to the corner on the opposite side of the plate (e.g., to corner 1 in the situation depicted on Fig. 4a). E1 started to call the UMO by saying “Come here!” and continuously encouraged the UMO to go there. When the UMO went to E1, she petted and praised the UMO. Then, E1 gave the “Stay!” command to the UMO and she went to the opposite side (e.g., corner 2 on Fig. 4a); during this the UMO did not move. Upon arriving, E1 started to call the UMO again. The Come-stay routine was repeated overall three times. After the third time, E1 again gave the “Stay!” command to the UMO, and then went to the plate, picked it up and called the UMO’s attention by saying “Look!”. She went back to the UMO and gave the “Fetch it!” command to the UMO while pointing toward the plate. The UMO went to the plate, picked it up and took it to E1; E1 continuously encouraged the UMO in the meantime. The UMO could move the plate by having magnets on the front of the UMO and metal sheets on the sides of the plate. When the UMO arrived to E1, she petted and praised the UMO and took the plate off. Then, E1 moved to the opposite corner of the room while continuously encouraging the UMO to go with her. We repeated the Fetch-it routine one more time. At the end, E1 and the UMO left the room together through Door A; E1 continuously encouraged the UMO to go with her.

In the Non-interactive UMO group, both E1 and the UMO carried out the same behaviour as in the Interactive UMO group, but the UMO did it in reverse order and E1 never turned toward the UMO while giving the commands/encouragement. Thus, although the UMO had the same animate and goal-directed behaviour, it did not engage in a social interaction with E1. While E1 was carrying out the Come-stay routine (the maximum of five times), the UMO picked up and put down the plate. While giving the “Come here!” and “Stay” commands, E1 was never turned toward the UMO, and she petted and praised an imaginary UMO (keeping similar timings as with the UMO in the other group). The UMO picked up the plate, went to the block on the opposite side and put it down by pushing the plate to the side of the block. Then, it moved without the plate to the opposite block, stayed there for 2–3 s and then moved back, picked the plate up and took it to the other block. In case the UMO could not put the plate down, it carried out the motion with the plate on it. At the end of this routine, the UMO moved to the corner on the opposite side of the room starting the “come-stay routine”, and E1 started the Fetch-it routine. In case the plate was still on the UMO, E1 took it off without either the UMO or E1 stopping. While giving the “Look!” and “Fetch it!” commands, and while giving encouragement, E1 did not look at the UMO. At the end, E1 left the room through Door A (while giving encouragement to an imaginary UMO), and a few seconds later the UMO went out of the room as well.

In the Mechanistic UMO group, the UMO moved in a circular route around the room, and E1 moved around on a winding route, while repeating the same commands, encouragements, and praises as in the other groups without looking at the UMO. After 90 s (measured by E1 on a stopwatch), E1 left the room when she reached the door on her route, and the UMO went out the room next, when it reached Door A on its route. Thus, here the UMO displayed a repetitive, mechanistic behaviour independently of E1.

From the corridor, either E1 or E2 picked up the UMO and placed it in the adjacent room. E1 returned to the room, and the owner and the dog left the room for a 2–3 min break before the next phase.

#### Jealousy-evoking phase

E1 gave the instructions to the owner during the break, emphasising again that they can stop the trials or the test at any time. Owners were instructed to interact only with the test partner and ignore the subject dog completely; however, in case they assessed that the subject dog may harm the other dog or the owner, or the test partner would harm the subject dog (even by accident), they could attend to the subject dog and stop the test at their own discretion (E1 and E2 could also intervene if needed). If the first test partner was the other dog, we simply asked the owner to behave naturally with the dog, as they would do in an everyday situation, but we instructed them not to call in the dog and say its name, do not give commands to the dog or play with them (thus mainly pet and talk to the dog). In case the first test partner was the UMO or the magazine, we instructed the owner to behave in a way as they do with the other dog. Following the first trial, we instructed the owner to behave the same way with the other two test partners, for example, read aloud from the magazine if they talked to the other dog/UMO using the same tone and words, or move around with the magazine if they moved in the first trial, and putting it on the ground.

In the case of the UMO and the magazine, the test partner was placed in the room before the dog entered, whereas in the case of the other dog from the household, the two dogs entered the room at the same time led by the owner on leash. E1 entered the room with the owner and the dog(s), and left the room after a few seconds; upon entering the room, the owner took the dog(s) off leash. After E1 left the room, the owner counted until ten while ignoring both the subject dog and the test partner. Following this, they started to interact with the test partner. Regardless of the group, the UMO moved away from its current location in every 20 s (owners were notified before the condition that the UMO will move sometimes and that they can continue interacting with it while it is moving and after it stops).

Each trial lasted for 90 s from the moment the owner started to interact with the test partner. The time was measured by E1 on a stopwatch, and she entered the room again when the time elapsed. The owner and the dog(s) left the room with E1 for a 1–2 min break during which the owner could pet and talk to the subject dog. All dogs encountered all test partners (three trials); the order of the test partners was counterbalanced between dogs.

### Behaviour and data analyses

Behaviour of dogs was coded using Solomon Coder version 19.08.02 (https://solomon.andraspeter.com/), and statistical analyses were carried out using R version 4.2.2^31^ in RStudio version 2023.03.0 Build 386^32^. Inter-coder reliability was assessed on a random subsample of dogs (15% of subjects). For duration data, we exported all coding sheets and compared the agreement between coders, five frames per second, using Cohen’s kappa. For frequency data, we carried out Spearman correlation to analyse the agreement.

In the Observation phase, we coded dogs’ looking duration toward E1, the UMO and the Interaction (only relevant to assess in the Interactive UMO group, but was needed because the dog did not look only at the UMO or only at E1 in those frames), from the moment E1 started to move until the UMO left the room. Inter-coder reliability indicated acceptable agreement (mean κ ± SD = 0.773 ± 0.063). For the analysis, we used the summed looking duration toward E1, the UMO and the Interaction, using the percentage of looking at the demonstration. This was necessary, because looking at the interaction could only be defined in one of the groups, and thus led to a basic difference in behaviour coding of the three groups. Considering that with this analysis we only aimed to confirm whether dogs looked at the demonstration long enough to obtain information/experience necessary for the next phase, we suggest that using the summed data is appropriate here. We assessed whether looking duration toward the demonstration differed in the three groups while controlling for the UMO’s motion in the Introduction phase (circular vs. winding route). We used linear model (LM; “stats” package; “lmtest” package^33^); residuals of the model had normal distribution (Kolmogorov Smirnov test: D = 0.091, *p* = 0.673). We compared the model containing the test predictor and control variable with the reduced model (containing only the latter); based on log-likelihood ratio test (LRT).

In the Jealousy-evoking phase, we assessed the duration of (1) looking at the owner, test partner and the interaction (head orientation); (2) body orientation toward the owner, test partner, and the interaction (orientation of the body itself without the head); (3) touching (physical contact with) the owner, test partner or the interaction (touching both at the same time); (4) being near the owner (within 0.5 m); and (5) moving toward or in parallel with the owner, test partner, and moving toward the interaction. Inter-coder reliability was acceptable for the duration of looking (mean κ ± SD = 0.731 ± 0.061), body orientation (mean κ ± SD = 0.726 ± 0.120), and being near the owner (mean κ ± SD = 0.897 ± 0.054) data. Due to the low reliability, we excluded the data of touching (mean κ ± SD = 0.576 ± 0.111) and moving toward/in parallel with the owner, test partner or interaction (mean κ ± SD = 0.500 ± 0.187).

We performed a principal component analysis (PCA) (“psych” and “GPArotation” packages^34,35^) on the above behaviours with oblimin rotation (in Abdai et al.^6^, the same behaviours were included in the PCA analysis). We retained three components based on the Horn’ parallel analysis (“paran” package^36^): PC1 – Interaction-related behaviour; PC2 – Owner-related behaviour; PC3 - Test partner-related behaviour (eigenvalues: PC1, 1.891; PC2, 1.570; PC3, 1.378). For each component, we calculated Cronbach’s alpha values; all had sufficient reliability (see Table 1). Based on the components, we calculated scores for each dog, which were analysed using linear mixed models (LMM, “lme4” package^37^). Residuals of the models analysing the Owner-related (Kolmogorov-Smirnov test: D = 0.062; *p* = 0.489), and Interaction-related behaviours (D = 0.053; *p* = 0.703) had normal distribution. Following Box-Cox transformation (lambda = -1; “MASS” package^38^), the residuals of the model analysing Test partner-related behaviour was also normally distributed (D = 0.041; *p* = 0.926). In all models, we entered the fixed main effects and all two-way interactions of Group (Interactive UMO vs Non-interactive UMO vs Mechanistic UMO), Condition (Dog, UMO, Reading), and Trial number (Trial 1 vs 2 vs 3), as well as the identification number of the subject as random effect to control for repeated measures. We carried out backward model selection using the drop1 function (“stats” package) based on LRT. For non-significant variables, we report the result of the LRT before exclusion from the model. In the case of significant variables, we carried out pairwise comparison with Tukey correction (“emmeans” package^39^).

We also measured the frequency of (1) interrupting the owner-test partner interaction (moving between them); (2) vocalization (barking, growling, and whining); (3) snapping at the test partner; and (4) stress-related behaviours (e.g., lip licking and yawning). Inter-coder reliability was acceptable for the frequency of interrupting the owner-test partner interaction (ρ = 0.797, *p* < 0.001), vocalization (ρ = 0.657, *p* < 0.001), and snapping (ρ = 0.946, *p* < 0.001). However, inter-coder reliability yielded insufficient reliability in the case of stress-related behaviours (ρ = 0.089, *p* = 0.656).

Based on the AIC values (model comparison with ANOVA; the model with the lowest AIC value was kept, the model was evaluated as better if ΔAIC > 2), the negative binomial distribution fit best the frequency of interruption (AIC = 541.37), vocalization (AIC = 584.61) and snapping (AIC = 410.07). Thus, we applied generalized linear mixed model (GLMM) with negative binomial distribution (“lme4” package^37^) to analyse the data. The fixed and random variables included in the model, and data analysis procedure were the same as described in the case of the PCA scores.

## Electronic supplementary material

Below is the link to the electronic supplementary material.

Supplementary Material 1

Supplementary Material 2

## Data Availability

All data are available as Supplementary material.

## References

[1] Mize, K. D. & Jones, N. A. Infant physiological and behavioral responses to loss of maternal attention to a social-rival. *Int. J. Psychophysiol.* **83**, 16–23 (2012). 21989365 10.1016/j.ijpsycho.2011.09.018

[2] Hart, S. L. Proximal foundations of jealousy: expectations of exclusivity in the infant’s first year of life. *Emot. Rev.* **8**, 358–366 (2016). 28232851 10.1177/1754073915615431 PMC5302134

[3] Parrot, W. G. & Smith, R. H. Distinguishing the experiences of envy and jealousy. *J. Pers. Soc. Psychol.* **64**, 906–920 (1993). 8326472 10.1037//0022-3514.64.6.906

[4] Prato-Previde, E. & Valsecchi, P. What is it like to be a jealous dog? *Anim. Sentience*. **3**, 16 (2018).

[5] Abdai, J. & Miklósi, Á. Displaying jealous behavior versus experiencing jealousy. *Anim. Sentience*. **3**, 21 (2018).

[6] Abdai, J., Baño Terencio, C. & Pérez Fraga, P. Miklósi, Á. Investigating jealous behaviour in dogs. *Sci. Rep.* **8**, 8911 (2018). 29891847 10.1038/s41598-018-27251-1 PMC5996015

[7] Hart, S. & Carrington, H. Jealousy in 6-month-old infants. *Infancy* **3**, 395–402 (2002). 33451216 10.1207/S15327078IN0303_6

[8] Mize, K. D., Pineda, M., Blau, A. K., Marsh, K. & Jones, N. A. Infant physiological and behavioral responses to a jealousy provoking condition. *Infancy* **19**, 338–348 (2014).

[9] Prato-Previde, E. & Valsecchi, P. The immaterial cord: the dog-human attachment bond. in The Social Dog (eds Kaminski, J. & Marshall-Pescini, S.) 165–189 (Academic, Boston, (2014).

[10] Topál, J., Miklósi, Á. & Csányi, V. Attachment behaviour in the dogs (Canis familiaris): a new application of the Ainsworth’s (1969) strange Situation Test. *J. Comp. Psychol.* **112**, 219–229 (1998). 9770312 10.1037/0735-7036.112.3.219

[11] Bastos, A. P. M., Neilands, P. D., Hassall, R. S., Lim, B. C. & Taylor, A. H. Dogs mentally represent jealousy-inducing social interactions. *Psychol. Sci.* **32**, 646–654 (2021). 33825583 10.1177/0956797620979149

[12] Harris, C. R. & Prouvost, C. Jealousy in dogs. *PLoS One*. **9**, e94597 (2014). 25054800 10.1371/journal.pone.0094597 PMC4108309

[13] Prato-Previde, E., Nicotra, V., Fusar Poli, S., Pelosi, A. & Valsecchi, P. Do dogs exhibit jealous behaviors when their owner attends to their companion dog? *Anim. Cogn.* **21**, 703–713 (2018). 30051326 10.1007/s10071-018-1204-0

[14] Prato-Previde, E., Nicotra, V., Pelosi, A. & Valsecchi, P. Pet dogs’ behavior when the owner and an unfamiliar person attend to a faux rival. *PLoS One*. **13**, e0194577 (2018). 29668684 10.1371/journal.pone.0194577 PMC5905953

[15] Karl, S., Anderle, K., Völter, C. J. & Virányi, Z. Pet dogs’ behavioural reaction to their caregiver’s interactions with a third party: join in or interrupt? *Animals* **12**, 1574 (2022). 35739910 10.3390/ani12121574 PMC9219478

[16] Hart, S. L. Pathways of development. in Jealousy in Infants: Laboratory Research on Differential Treatment (Springer, New York, NY, (2015).

[17] Abdai, J. et al. Individual recognition and long-term memory of inanimate interactive agents and humans in dogs. *Anim. Cogn.* **25**, 1427–1442 (2022). 35513745 10.1007/s10071-022-01624-6 PMC9652224

[18] Abdai, J., Gergely, A., Petró, E., Topál, J. & Miklósi, Á. An investigation on social representations: Inanimate agent can mislead dogs (Canis familiaris) in a food choice task. *PLoS One* **10**, e0134575 (2015). 10.1371/journal.pone.0134575 PMC4524664 26241747

[19] Gergely, A. et al. Dogs rapidly develop socially competent behaviour while interacting with a contingently responding self-propelled object. *Anim. Behav.* **108**, 137–144 (2015).

[20] Capitain, S., Miklósi, Á. & Abdai, J. Influence of reward and location on dogs’ behaviour toward an interactive artificial agent. *Sci. Rep.* **13**, 1093 (2023). 36658170 10.1038/s41598-023-27930-8 PMC9852237

[21] Abdai, J., Uccheddu, S., Gácsi, M. & Miklósi, Á. Exploring the advantages of using artificial agents to investigate animacy perception in cats and dogs. *Bioinspir. Biomim.* **17**, 065009 (2022). 10.1088/1748-3190/ac93d9 36130608

[22] Abdai, J., Baño Terencio, C. & Miklósi, Á. Novel approach to study the perception of animacy in dogs. *PLoS One*. **12**, e0177010 (2017). 28472117 10.1371/journal.pone.0177010 PMC5417633

[23] Gergely, A., Compton, A. B., Newberry, R. C. & Miklósi, Á. Social interaction with an ‘Unidentified moving object’ elicits A-not-B error in domestic dogs. *PLoS One*. **11**, e0151600 (2016). 27073867 10.1371/journal.pone.0151600 PMC4830451

[24] Cook, P., Prichard, A., Spivak, M. & Berns, G. S. Jealousy in dogs? Evidence from brain imaging. *Anim. Sentience*. **22**, 1 (2018).

[25] Bräuer, J. & Amici, F. Fake or not: two prerequisites for jealousy. *Anim. Sentience*. **3**, 18 (2018).

[26] Vonk, J. Researchers, not dogs, lack control in an experiment on jealousy. *Anim. Sentience*. **3**, 2 (2018).

[27] Serpell, J. A. Jealousy? Or just hostility toward other dogs? The risks of jumping to conclusions. *Anim. Sentience*. **3**, 13 (2018).

[28] Morris, P. H., Doe, C. & Godsell, E. Secondary emotions in non-primate species? Behavioural reports and subjective claims by animal owners. *Cogn. Emot.* **22**, 3–20 (2008).

[29] Gergely, A., Petró, E., Topál, J. & Miklosi, A. What are you or who are you? The emergence of social interaction between dog and an unidentified moving object (UMO). *PLoS One*. **8**, e72727 (2013). 24015272 10.1371/journal.pone.0072727 PMC3755977

[30] Csibra, G. Goal attribution to inanimate agents by 6.5-month-old infants. *Cognition* **107**, 705–717 (2008). 17869235 10.1016/j.cognition.2007.08.001

[31] R Core Team. R: A language and environment for statistical computing. Preprint at (2022). https://www.r-project.org/

[32] Posit team. RStudio: Integrated development environment for R. Preprint at (2023). http://www.posit.co/

[33] Zeileis, A. & Hothorn, T. Diagnostic checking in regression relationships. *R News*. **2**, 7–10 (2002).

[34] Bernaards, C. A. & Jennrich, R. I. Gradient projection algorithms and software for arbitrary rotation criteria in factor analysis. *Educ. Psychol. Meas.* **65**, 676–696 (2005).

[35] Revelle, W. psych: Procedures for psychological, psychometric, and personality research. Preprint at (2022). https://cran.r-project.org/package=psych

[36] Dinno, A. & paran Horn’s test of principal components/factors. Preprint at (2018). https://cran.r-project.org/package=paran

[37] Bates, D., Maechler, M., Bolker, B. & Walker, S. Fitting linear mixed-effects models using lme4. *J. Stat. Softw.* **67**, 1–48 (2015).

[38] Venables, W. N. & Ripley, B. D. *Modern Applied Statistics with S* (Springer, 2002).

[39] Lenth, R. V. & emmeans Estimated marginal means, aka least-squares means. Preprint at (2022). https://cran.r-project.org/package=emmeans

